# CTCF deletion alters the pluripotency and DNA methylation profile of human iPSCs

**DOI:** 10.3389/fcell.2023.1302448

**Published:** 2023-11-30

**Authors:** Deepika Puri, Catharina Maaßen, Monica Varona Baranda, Kira Zeevaert, Lena Hahnfeld, Annika Hauser, Giulia Fornero, Mohamed H. Elsafi Mabrouk, Wolfgang Wagner

**Affiliations:** ^1^ Institute for Stem Cell Biology, RWTH Aachen University Medical School, Aachen, Germany; ^2^ Helmholtz-Institute for Biomedical Engineering, RWTH Aachen University Medical School, Aachen, Germany

**Keywords:** IPSC, CTCF, DNA methylation, pluripotency, differentiation, CRISPR, stem cells

## Abstract

Pluripotent stem cells are characterized by their differentiation potential toward endoderm, mesoderm, and ectoderm. However, it is still largely unclear how these cell-fate decisions are mediated by epigenetic mechanisms. In this study, we explored the relevance of CCCTC-binding factor (CTCF), a zinc finger-containing DNA-binding protein, which mediates long-range chromatin organization, for directed cell-fate determination. We generated human induced pluripotent stem cell (iPSC) lines with deletions in the protein-coding region in exon 3 of CTCF, resulting in shorter transcripts and overall reduced protein expression. Chromatin immunoprecipitation showed a considerable loss of CTCF binding to target sites. The CTCF deletions resulted in slower growth and modest global changes in gene expression, with downregulation of a subset of pluripotency-associated genes and neuroectodermal genes. CTCF deletion also evoked DNA methylation changes, which were moderately associated with differential gene expression. Notably, CTCF-deletions lead to upregulation of endo-mesodermal associated marker genes and epigenetic signatures, whereas ectodermal differentiation was defective. These results indicate that CTCF plays an important role in the maintenance of pluripotency and differentiation, especially towards ectodermal lineages.

## 1 Introduction

Differentiation of induced pluripotent stem cells (iPSCs) toward specific cell types remains a challenge and the underlying mechanisms that control cell-fate decisions are so far hardly understood. During the reprogramming of somatic cells into iPSCs and their lineage-specific differentiation, extensive epigenetic remodeling occurs. Notably, studies have shown that while the tissue source used to generate the iPSCs may affect the propensity of targeted differentiation due to epigenetic memory (Scesa et al., 2021), this can be overcome by altering the epigenetic state of the iPSCs to improve the efficiency and fidelity of iPSC differentiation (Kim et al., 2010; Buckberry et al., 2023). The concerted and genome-wide nature of these epigenetic modifications suggests that differentiation and lineage commitment decisions may be mediated by intra- and inter-chromosomal interactions that regulate the 3D chromatin architecture and thereby orchestrate gene expression patterns. Thus, proteins that govern chromatin structure may play a central role in cell fate decisions towards specific germ layers and terminally differentiated cells.

CTCF is an insulator protein that regulates long-range chromatin interfaces, and enhancer-promoter interactions to govern gene expression and cell fate (Dehingia et al., 2022). CTCF interacts with cohesins and occupies CCCTC regions in the genome to form distinct topologically associated domains (TADs) that govern chromatin organization (Szabo et al., 2019). During reprogramming, CTCF acts as an insulator to suppress somatic genes and facilitate the expression of pluripotency genes (Song et al., 2022). A recent study has also implicated CTCF as a barrier to the reprogramming of embryonic stem cells back into a more totipotent 2C-like stage, indicating an important role in attaining and maintaining the pluripotent stem cell state (Olbrich et al., 2021). However, the precise role of CTCF in iPSC pluripotency and differentiation potential is largely unknown. Some insights can be obtained from mouse genetic studies, that have revealed that CTCF homozygous null mice are embryonic lethal, indicating an essential role during embryogenesis and development (Wan et al., 2008; Moore et al., 2012). By using acute depletion strategies such as the auxin-inducible degron (AID) system, studies in murine embryonic stem cells (mESCs) have characterized CTCF-depleted cells and reported a modestly changed transcription profile and a more significant role in enhancer-promoter interaction-driven changes in TADs (Nora et al., 2017; Kubo et al., 2021). However, these studies do not address the role of CTCF during stem cell differentiation.

In addition to the functional heterogeneity of different CTCF splice variants (Kim et al., 2015; Li et al., 2019) the function of CTCF also seems to depend on DNA methylation at the binding site, which harbors a CG dinucleotide (CpG site) that can become methylated. While some studies indicate that CTCF binding to target sites negatively correlates with DNA methylation (Bell and Felsenfeld, 2000; Kanduri et al., 2000), other reports suggest surprising plasticity between DNA methylation and CTCF enrichment, governed by cell type, genomic location, and DNA methylation levels (Wang et al., 2012). Thus, it can be anticipated that interaction of CTCF occupancy and DNA methylation contribute to cell-fate decisions in iPSCs.

In this study, we generated human iPSC lines with an N terminal CTCF deletion (CTCF-del), resulting in shorter transcripts and reduced protein expression. Notably, these iPSC lines revealed altered pluripotency and differentiation potential. Additionally, our study showed that while reduction of CTCF did not result in extensive changes in DNA methylation patterns, CTCF might affect DNA methylation and gene expression at specific targets, pointing to a more nuanced role in regulating cell fate determination.

## 2 Materials and methods

### 2.1 Cell culture

We used three human iPSC lines UKAi009-A (WT 102), UKAi010-A (WT 104), and UKAi011-A (WT 106) generated from bone marrow-derived mesenchymal stromal cells (Goetzke et al., 2018). All samples were taken after informed and written consent using guidelines approved by the Ethics Committee for the Use of Human Subjects at the University of Aachen (permit number: EK128/09) and methods were performed in accordance with the relevant guidelines and regulations. The iPSC lines were cultured on tissue culture plastic coated with vitronectin (0.5 μg/cm^2^; Stemcell Technologies) in StemMACS iPS-Brew XF (Miltenyi Biotec GmbH). We used the STEMdiff Trilineage Differentiation Kit (Stemcell Technologies) for directed differentiation towards endodermal, mesodermal, and ectodermal lineage, following manufacturers’ protocols.

### 2.2 CRISPR-Cas9 mediated CTCF deletion

Guide RNAs (gRNA) were designed to target the start codon of CTCF in exon 3 (Supplementary Table S1). To transfect the ribonucleoprotein (RNP) into the WT102 iPSCs, Alt-R CRISPR/Cas9 crRNA, Alt-R CRISPR/Cas9 tracrRNA (IDT, 237-185745), and Alt-R HiFi Sp. Cas9 Nuclease (IDT, 237185746) were used. The CrRNA and tracrRNA were resuspended in IDT duplex buffer, the crRNA-tracrRNA complex was produced. The complex was incubated with HiFi Sp. Cas9 to make the RNP, which was transfected into iPSCs using the NEON transfection system (Thermo Fischer Scientific) using standard protocols. Transfected cells were grown on laminin-coated tissue culture plates and murine embryonal fibroblasts (MEFs). Single colonies were isolated, expanded and tested for loss of CTCF. In total, over 70 clones were picked and screened via PCR, and about 10 clones were analyzed via Sanger sequencing (Eurofins Scientific). Finally, two stable cell lines (CTCF-del 50 and CTCF-del 55) were used for further experiments.

### 2.3 Growth curve

An equal number of iPSCs (100,000 cells; WT 102 and syngeneic CTCF-del 50 and 55) were plated on vitronectin-coated tissue culture plates. For cell counting, cells were dissociated with Accutase (Stemcell technologies) and harvested by centrifugation. Single cells were diluted with Trypan blue (Sigma Aldrich) and counted using the Countess 3 Automated Cell Counter (ThermoFisher Scientific). Cells were counted daily for 6 days and growth curves were plotted.

### 2.4 Western blot

Cells were harvested and total cell lysates were prepared in RIPA buffer (10 mM NaF, 1 mM Na3VO4, and 7 × complete mini protease inhibitor). Bradford assay was used to determine protein concentration. 20–30 μg of protein was incubated with 4× SDS Protein Sample Buffer (50 mM Tris/HCl pH 6.8, 2% SDS, 0.01% Bromophenol blue, 2.5% β-mercaptoethanol and 10% glycerol) for 5 min at 99°C and separated in 12% Mini-PROTEAN TGX Precast Protein Gels (Bio-Rad). The gels were transferred onto polyvinylidene fluoride (PVDF; Merck Millipore) membranes, which were blocked in 4% w/v bovine serum albumin (BSA) and incubated with primary antibodies as mentioned in (Supplementary Table S2) in blocking solution overnight at 4°C. The membranes were further incubated with secondary antibodies (Supplementary Table S2) for 1 h at RT and protein bands were visualized using a ChemiDoc XRS + (Bio-Rad Laboratories).

### 2.5 Immunostaining

Cells were fixed with 4% paraformaldehyde (PFA) for 20 min and permeabilized with PBS containing 1% w/v BSA and 0.1% v/v Triton X-100 (Bio-Rad) for 30 min, followed by overnight incubation at 4°C with primary antibodies against CTCF, OCT4 and PAX6 (Supplementary Table S2). Secondary antibody (Supplementary Table S2) staining was done at RT for 1 h. Samples were then counterstained with DAPI (10 ng/mL) for 15 min at RT in the dark. 2D samples were imaged using an LSM 700 confocal microscope (Carl Zeiss) using ×20 objective or ×10 objective.

### 2.6 Semi-quantitative reverse transcriptase PCR

Total RNA was isolated from undifferentiated and differentiated cells using the NucleoSpin RNA Plus Kit (Macherey-Nagel), quantified with a NanoDrop ND-2000 spectrophotometer (Thermo Scientific) and converted into cDNA using the High-Capacity cDNA Reverse Transcription Kit (Applied Biosystems). The cDNA was either amplified using endpoint PCR or semi-quantitative reverse-transcriptase PCR (RT-qPCR) using Power SYBR Green PCR Master Mix (Applied Biosystems) and gene-specific primers in a StepOnePlus machine (Applied Biosystems, Waltham) using primers described in (Supplementary Table S1).

### 2.7 Gene expression analysis

Library preparation (QuantSeq 3′-mRNA) and RNA-sequencing were performed by Life and Brain company (Bonn, Germany) on a NovaSeq 6000 sequencer (100 bp/read). Quality of FASTQ files was quantified using FastQC, and adapter sequences and low-quality reads were trimmed using Trim Galore. Alignment of the reads was done using STAR (hg38 genome build) and transcript quantification was performed using Salmon. The resulting count matrices were normalized by size factor and dispersion using the DESeq2 package in R (Love et al., 2014). Differential gene expression analysis between CTCF-del (CTCF-del 50 and CTCF-del 55) and wildtype samples (WT 102: 2 replicates, WT 104 and WT 106) was performed using Negative Binomial GLM fitting and Wald-test, and *p*-values were adjusted with the Benjamini–Hochberg procedure. Genes with adjusted *p*-value <0.05 and log_2_ fold change >1 were considered as differentially expressed. Pathway activation was inferred with the PROGENy package (Schubert et al., 2018) using top 500 pathway responsive genes. List of signature genes for each germ layer (ectoderm, endoderm and mesoderm) were obtained from previously published results (Schmidt et al., 2023; Zeevaert et al., 2023). Prediction of spatial gene expression of CTCF was carried out using the computation model of spatial gene expression of wildtype iPSCs colonies (Mabrouk et al., 2022). The model depends on immunofluorescence images of a small number of markers to guide the spatial reconstruction of gene expression patterns of all sequenced genes using optimal transport algorithm (Nitzan et al., 2019).

### 2.8 Chromatin immunoprecipitation

iPSCs were fixed with 1% formaldehyde and fractionated using the Covaris M220 using Peak power 75W, Duty factor 5%, Cycle/burst 200 at 6° for 25 min to obtain chromatin fractions ranging from 200 bp-1kb. Immunoprecipitation was performed with the CTCF antibody (Supplementary Table S2) using the ChIP-IT Express Chromatin Immunoprecipitation Kit (Activ Motif, #53008) using standard protocols. The purified DNA was sequenced at the Cologne Centre for Genomics.

The Nextflow nf-core/chipseq pipeline (v2.0.0; https://doi.org/10.5281/zenodo.7139814) was used for the analysis of chromatin immunoprecipitation sequencing (ChIP-seq) data together with Singularity containers. The trimmed reads were mapped to the Ensembl human reference genome GRCh38, and further analysis was carried out with default parameters. In brief, the pipeline performed quality control of the raw reads (FASTQC v0.11.9), quality and adapter trimming (Trim Galore! v0.6.7), read alignment (BWA v0.7.17-r1188), duplicate marking and filtering (picard v2.27.4-SNAPSHOT, SAMtools v1.15.1, BEDTools v2.30.0, BAMTools v2.5.2), generation of normalized coverage track files (BEDTools v2.30.0, ucsc_bedGraphToBigWig v377), narrow peak calling (MACS2 v2.2.7.1), annotation of the peaks relative to known genomic features (HOMER v4.11), creation of consensus peak set (BEDTools v2.30.0, subread_featureCounts v2.0.1), differential binding analysis (DESeq2—deseq2_qc.r script), and extensive library QC (picard v2.27.4-SNAPSHOT, Preseq v3.1.1, deepTools v3.5.1, phantompeakqualtools v1.1.2, MultiQC v1.13). After alignment, technical replicates were merged, and downstream analysis was performed on these merged data. Differential binding analysis was performed using THOR (v0.13.2; https://reg-gen.readthedocs.io/en/latest/thor/introduction.html) and genomic regions were filtered for significant binding (“filter-THOR p-val 20”). The R package ChIPseeker https://bioconductor.org/packages/release/bioc/html/ChIPseeker.html was used to perform peak annotation and functional enrichment analysis.

### 2.9 Pyrosequencing analysis

500 ng genomic DNA from WT and CTCF-del cells was bisulfite converted overnight using the EZ DNA Methylation Kit (Zymo) and eluted in 20 μL elution buffer. PyroMark Assay Design 2.0 Software from Qiagen was used to design the primers (Supplementary Table S1). Target sequences were amplified with the PyroMark PCR Kit (Qiagen) with 2.5 mM Mg2 + and a primer concentration of 0.3 μM. Pyrosequencing was performed on a Q48 ID pyrosequencer (Qiagen). Pluripotency score was calculated based on DNA methylation at three specific CpGs: cg00661673, associated with the gene Palladin (*PALLD*); cg00933813, not associated with a specific gene; and cg21699252, associated with MYCN opposite strand (*MYCNOS*). The DNA methylation values were combined into a pluripotency score as described before (Schmidt et al., 2023).

### 2.10 DNA methylation analysis

Genomic DNA was isolated from WT and CTCF-del iPSCs using the NucleoSpin Tissue Kit (Macherey-Nagel) with the manufacturer’s instructions and quantified with a NanoDrop ND-2000 spectrophotometer (Thermo Scientific). 1.2 μg DNA was bisulfite converted and analyzed with Illumina Beadchip Epic Array at Life and Brain (Bonn, Germany). Raw IDAT files were used for quality control and ssNoob normalization (Triche et al., 2013) with the minfi R package (Aryee et al., 2014). Detection *p*-values were calculated with the SeSAMe R package (Zhou et al., 2018) and the pOOBAH approach. CpG sites on XY chromosomes, non-cg probes, probes with a detection *p*-value >0.05 in two or more samples, and probes flagged in Illumina epic manifest version b5 were removed. This reduced the number to 678,229 CpG sites, which were used for further analysis. CpG sites with a difference of mean beta values ≥ 0.2 were considered differentially methylated. For combining DNAm and gene expression data, we used the Illumina BeadChip annotation and merged the data by matching to Ensembl IDs, considering CpGs in all gene positions (promoter, UTR and gene body). The R packages ggplot2 and ComplexHeatmap were used for generating the figures. Lists of differentially methylated CpGs for each germ layer were obtained from a previous publication (Schmidt et al., 2023). The similarity in methylation status (hypermethylated, hypomethylated, or not differentially methylated) were compared using a t-test. The Epi-Pluri Score was calculated from the DNAm levels in cg23737055 (*ANKRD46*), cg22247240 (*C14orf115*), and cg13083810 (*POU5F1*) as described in (Lenz et al., 2015).

## 3 Results

### 3.1 Generation and characterization of CTCF-del human iPSC lines

**FIGURE 1:**
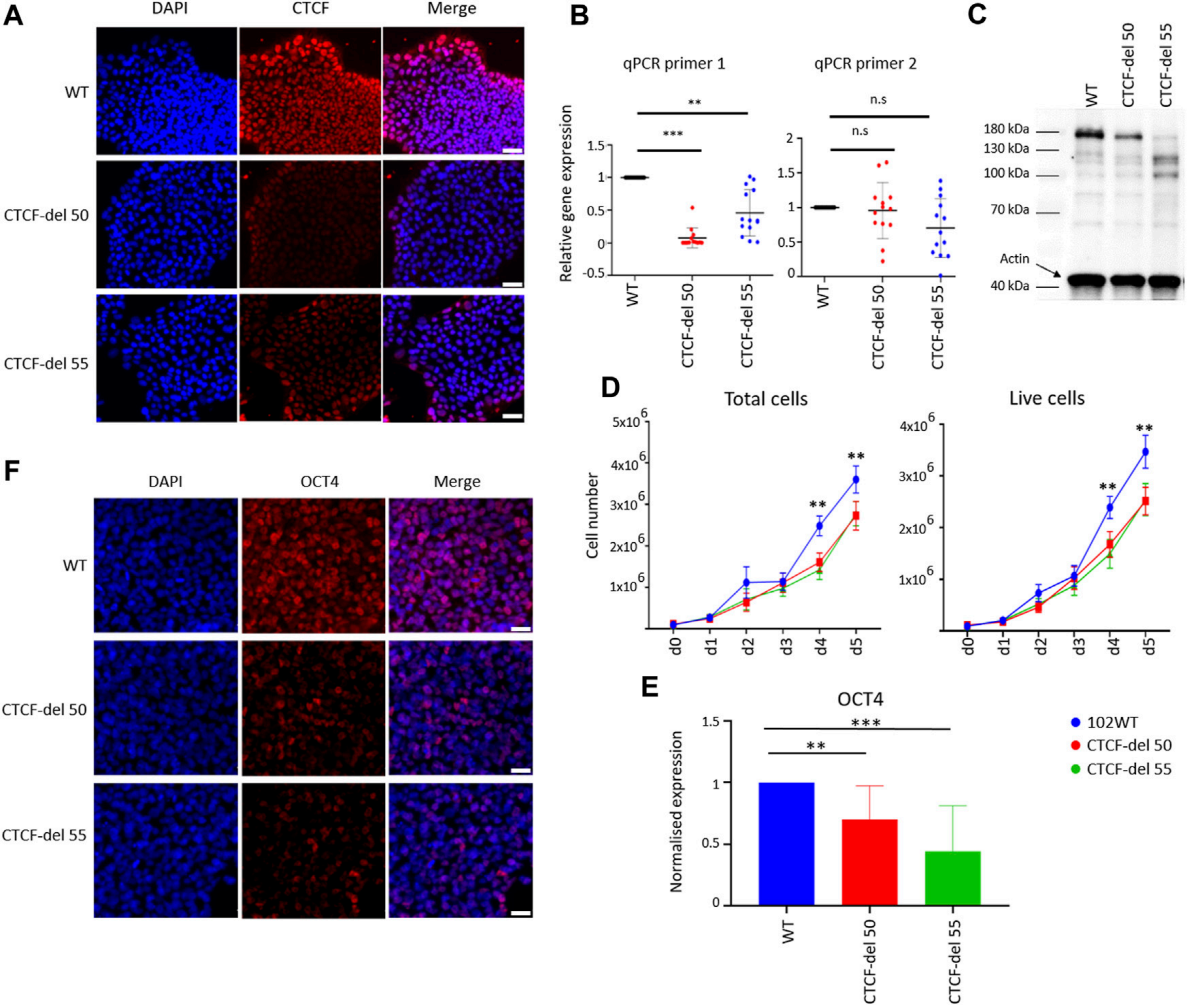
Characterization of CTCF-del iPSCs lines. **(A)** Immunofluorescence analysis shows reduction of CTCF in CTCF-del 50 and 55 clones compared to syngenic wild-type cells (WT). DAPI was used to stain the nuclei; Scale bar: 50 μm **(B)** RT-qPCR analysis validated a marked reduction of CTCF transcripts around the Cas9 deletion site (primer 1), whereas the transcript levels were less affected downstream of the deletion site (primer 2). The *Y*-axis represents the normalized expression to GAPDH. Paired t-tests were used to estimate statistical significance (***p-value <0.0005, **p-value <0.005; n = 4). **(C)** Western blot analysis indicates the CTCF protein levels are reduced in CTCF-del clone 50 and 55 compared to WT. Actin was used as loading control. **(D)** Growth curves demonstrate that CTCF-del clones proliferate slower than WT. Paired t tests were used to estimate significance (**p-value <0.005; n = 4). **(E)** qRT-PCR analysis of *OCT4* transcript levels (normalized to *GAPDH* and fold-change in comparison to WT; paired t-test **p-value <0.005, ***p-value <0.0005; n = 4). **(F)** Immunofluorescence analysis shows a reduction of OCT4 in CTCF-del 50 and 55 clones compared to WT. Nuclei stained with DAPI; Scale bar: 50 μm.

We generated two human iPSC lines with deletions in CTCF using CRISPR/Cas9 gene editing by targeting exon 3, which contains the translation start site (Suppleentary Figures S1A–C). Two guide RNAs were used spanning the translation start site, which led to a homozygous deletion of 534 bp, resulting in a frameshift mutation and a premature stop codon. The loss of the target region was confirmed by PCR and Sanger sequencing, and out of 70 clones analyzed, two (CTCF-del 50 and CTCF-del 55) were selected for further experiments (Supplementary Figures S1B,C). Immunofluorescence demonstrated a reduced expression of CTCF in both CTCF-deletion lines (Figure 1A). Notably, in wild-type (WT) iPSCs, the CTCF expression was higher at the borders of colonies as compared to the center—an area that also reveals higher expression of pluripotency markers (Mabrouk et al., 2022). This is in line with predictions of the spatial reconstruction of single-cell RNA sequencing data (Mabrouk et al., 2022) (Supplementary Figure S1D). Notably, the spatial organization of residual CTCF signals was also maintained in the CTCF-del lines, despite the markedly decreased expression levels. Furthermore, RT-qPCR analysis showed that the region surrounding the translation start site was hardly detected at the mRNA level, whereas expression of downstream sequences was still detected (Figure 1B). We also confirmed the lack of the full-length transcript by amplifying a region surrounding the translation start site from cDNA generated from WT and CTCF-del clones (Supplementary Figure S1E). Sanger sequencing of these amplicons further validated that CTCF deletions resulted in shorter transcripts without the initial transcription start site. Western blot also confirmed a reduction, but not a complete loss of CTCF at the protein level (Figure 1C). This suggests that while our CTCF-del clones may express some splice variants, the expression of CTCF was overall decreased and lacked the full-length transcript that may be relevant for CTCF function.

In comparison to wild-type (WT) iPSC lines, both the CTCF-del clones showed normal cellular morphology, without obvious differences over multiple passages (Supplementary Figure S1F). However, cell-counting assays showed reduced proliferation in CTCF-del clones (Figure 1D). Furthermore, CTCF-del cells showed reduced expression of the pluripotency protein OCT4 at RNA and protein levels (Figures 1E,F) indicating that loss of CTCF may alter the pluripotency of human iPSCs.

### 3.2 ChIP-Seq analysis of CTCF-del clones

**FIGURE 2:**
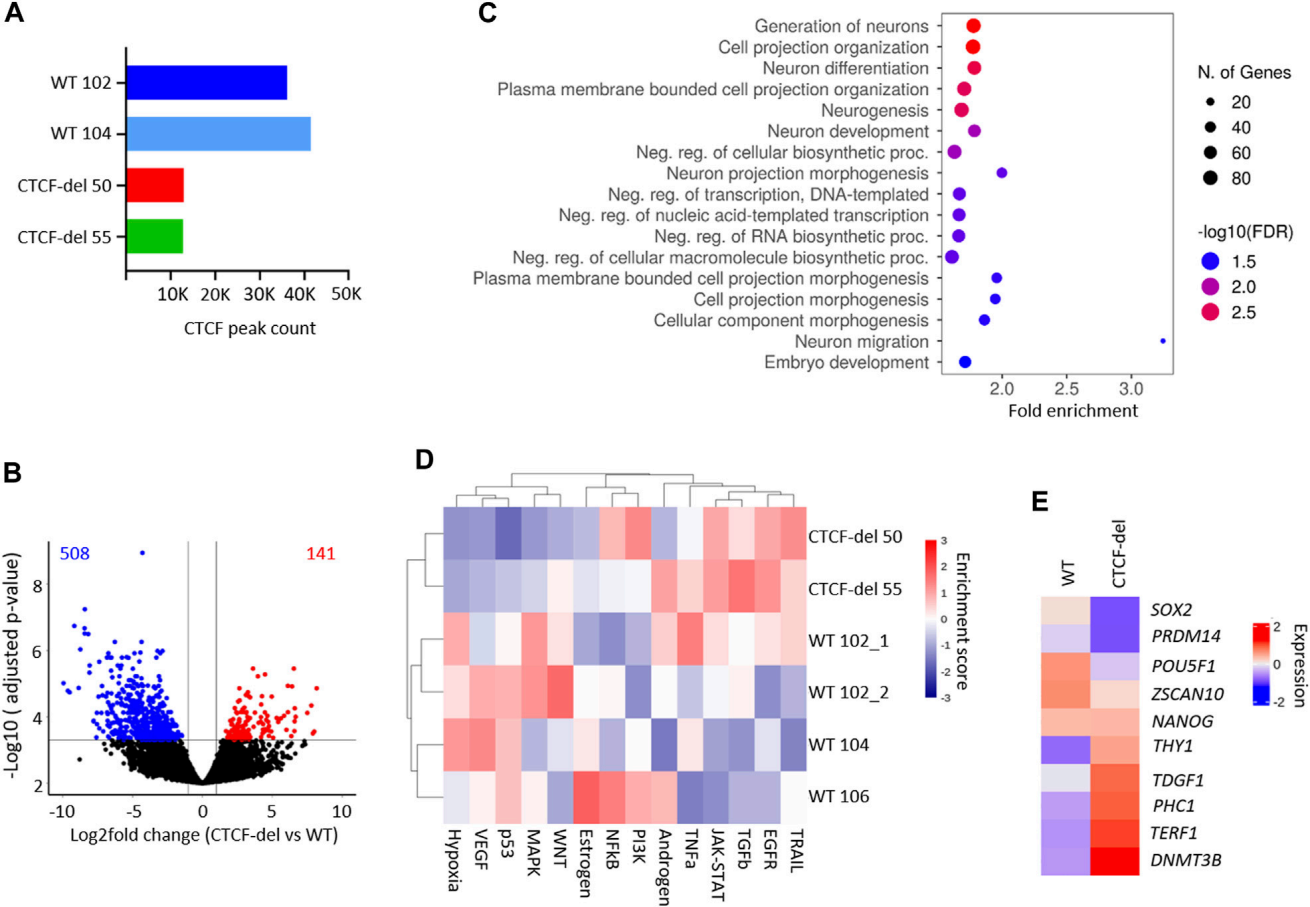
CTCF deletion alters the gene expression. **(A)** Total peak counts for CTCF ChIP-Seq in WT and CTCF-del iPSCs. CTCF-del results in reduced CTCF binding at target sites. **(B)** Volcano plot of RNA sequencing analysis of CTCF-del (n = 2) and WT (n = 4) iPSCs. 508 genes are significantly downregulated and 141 genes are significantly upregulated in CTCF-del (>2-fold change; adj. *p*-value < 0.05). **(C)** Gene set enrichment analysis of downregulated genes reveals significant enrichment in categories associated with transcription and neuronal development and differentiation. **(D)** Pathway analysis shows enrichment of TRAIL, EGFR, TGFb and JAK-STAT pathways and reduction of VEGF, MAPK, p53 and hypoxia pathways in CTCF-del *versus* WT iPSCs. **(E)** Normalized gene expression of 10 canonical marker genes for pluripotency in undifferentiated WT and CTCF-del cells.

To determine whether CTCF-del cells exhibit alterations in CTCF function, we performed ChIP-Seq in two WT iPSC clones and the two CTCF-del clones. In WT iPSCs, CTCF was enriched at 36,127 (WT 102) and 41,426 (WT 104) sites respectively, while in CTCF-del cells the CTCF enrichment was reduced to 13,000 (CTCF-del clone 50) and 12,817 sites (CTCF-del clone 55), respectively (Figure 2A). This indicates that while the CTCF-del clones may express some splice variants, our genetic deletion led to a 65%–70% reduction of CTCF binding at target sites. Differential analysis indicated that CTCF-del resulted in the loss of CTCF binding, largely from promoter regions, while other gene features show a similar distribution as in WT iPSCs (Supplementary Figure S2A).

### 3.3 Transcriptional analysis of CTCF-del clones

Previous studies suggested that acute depletion of CTCF in mESCs does not significantly affect the transcriptome (Nora et al., 2017; Kubo et al., 2021). To investigate how the deletions in CTCF affect gene expression profiles of our iPSC lines, we generated mRNA sequencing profiles of four WT iPSC lines and two CTCF-del lines. We observed that 141 genes were significantly upregulated and 508 genes were significantly downregulated in CTCF-del lines as compared to WT iPSC lines (>2-fold change; adj. *p*-value <0.05; Figure 2B and Supplementary Figure S2B). Among the most downregulated were genes involved in neuro-ectodermal determination and neural differentiation, such as *SIX3, MATR3,* and *PRTG* (Figure 2C, and Supplementary Figure S2B). This functional enrichment was also evident in gene ontology classification that revealed the enrichment of neuronal development and differentiation genes among the downregulated genes in CTCF-del (Figure 2C). Upregulated genes were enriched in oxidative phosphorylation, electron transport, metabolic and cell signaling, including the inhibitor for WNT, *NOTUM* (Supplementary Figures S2B,C). Pathway analysis revealed an upregulation of the TRAIL, EGFR, TGFb and JAK-STAT pathways and a downregulation of the MAPK, p53, VEGF and Hypoxia pathways (Figure 2D).

Since we observed reduced OCT4 expression in CTCF-del cells, we further investigated the expression of 10 canonical pluripotency genes (Schmidt et al., 2023; Zeevaert et al., 2023). We found a downregulation of *SOX2, PRDM14, POU5F1* (OCT4) and *ZSCAN10* while the other genes remained either unchanged or were upregulated (Figure 2E). Recent reports indicate that CTCF acts as a barrier for the reprogramming of stem cells into a totipotent 2C-like state (Olbrich et al., 2021), which is associated with an upregulation of genes such as *ZSCAN4* and repeats such as *M/HERVL*. We compared the expression levels of 2C-associated genes (Hendrickson et al., 2017) in our WT and CTCF-del lines. Other than *ZSCAN4, ALPPL2* and *CCNA1*, the 2C-associated genes remain repressed in CTCF del lines (Supplementary Figure S2D), indicating that the residual CTCF protein would still act as a barrier for 2C conversion.

### 3.4 DNA methylation analysis in CTCF-del cells

**FIGURE 3:**
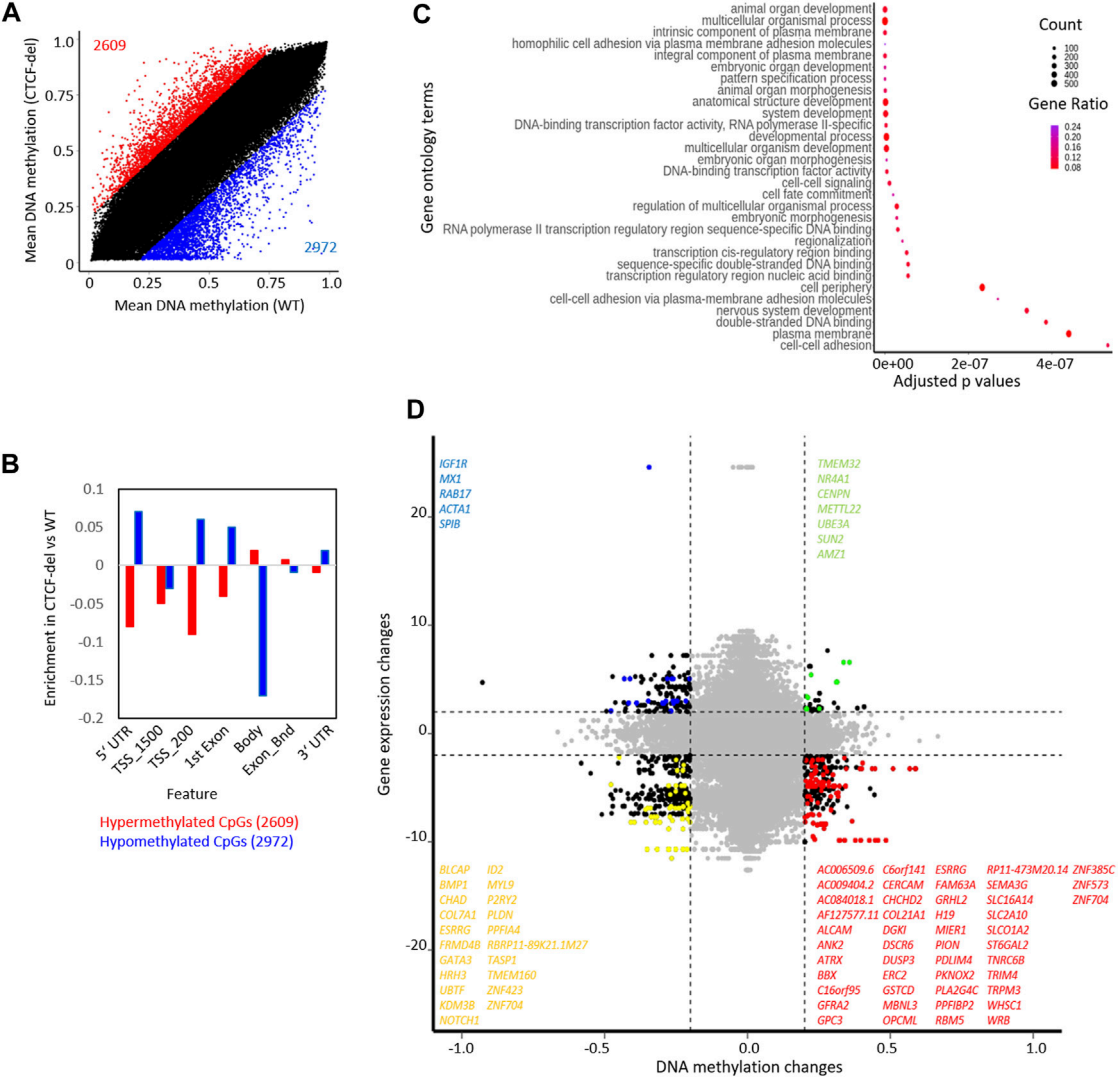
CTCF deletion alters the DNA methylation profile of hIPSCs. **(A)** CTCF deficiency causes global DNA methylation changes between CTCF-del (n = 2) and WT (n = 2) iPSCs. 2971 CpGs are significantly hypomethylated, and 2609 CpGs are significantly hypermethylated in CTCF-del iPSCs (difference in mean DNA methylation ≥20%). **(B)** Feature analysis for hypermethylated and hypomethylated CpGs in CTCF-del iPSCs (association of CpGs with gene regions were taken from the Illumina annotation; enrichment as calculated vs. all CpGs considered in the analysis). **(C)** Gene set enrichment analysis of hypomethylated CpGs demonstrates significant enrichment of associated genes in transcription, cell fate commitment, development, and cell adhesion. **(D)** Association of DNA methylation and corresponding gene expression in WT *versus* CTCF-del iPSCs. Only CpGs in promoter regions (TSS1500, TSS200) are considered (significance cut-offs as indicated above). Each dot represents a gene-CpG-pair (genes as well as CpGs might be duplicated). Representative significant genes are depicted.

The CTCF deletions might also directly interfere with the DNA methylation pattern, particularly because the CTCF binding sequence itself contains a CpG site. DNA methylation analysis of CTCF-del clones revealed significant (difference in mean DNA methylation ≥0.2) hypermethylation of 2609 CpGs and hypomethylation of 2972 CpGs compared to WT iPSCs (Figure 3A). Of the differentially methylated CpGs, 192 hypomethylated CpGs and 160 hypermethylated CpGs overlapped with CTCF ChIP-Seq peaks (Supplementary Figure S3A). The hypermethylated CpGs were enriched in the gene body of corresponding genes, whereas hypomethylated CpGs were associated with 5′ and 3′ UTRs and TSS-upstream regions (Figure 3B). While genes associated with hypermethylated CpGs did not cluster based on function, the genes associated with hypomethylated CpGs were involved in processes such as cell adhesion, transcription, and development (Figure 3C).

Our previous work indicated that DNA methylation during human aging was enriched at CTCF binding sites (Han et al., 2020; Franzen et al., 2021). Therefore, we wanted to explore how CTCF deletions influence this epigenetic process. Epigenetic age-predictions using the Horvath clock (Horvath, 2013) or the skin and blood clock (Horvath et al., 2018) suggested that all iPSC lines—including the CTCF-del lines—were predicted close to 0 years age (Supplementary Figure S3B). This is in line with the finding that age-associated DNA methylation patterns are generally reversed during reprogramming into the pluripotent state and may not depend on CTCF function.

Next, we investigated whether the gene expression changes correlated with changes in DNA methylation. Out of the 141 upregulated genes in CTCF-del, 5 genes were also associated with hypomethylated CpGs, and the 508 downregulated genes comprised 47 genes that were associated with hypermethylated CpGs (Figure 3D). Notably, the *H19* gene was downregulated and its promoter hypermethylated in CTCF-del clones (Supplementary Figure S3C), which is in line with the finding that CTCF binding is known to inhibit DNA methylation at the IGF2/H19 locus and mutations in the CTCF binding sites lead to hypermethylation of the locus and the downregulation of the gene (Schoenherr et al., 2003; Szabo et al., 2004). There was no change in the expression of *IGF2* suggesting that the regulation of this gene may be independent of CTCF.

### 3.5 Differentiation potential is impaired in CTCF-del iPSCs

**FIGURE 4:**
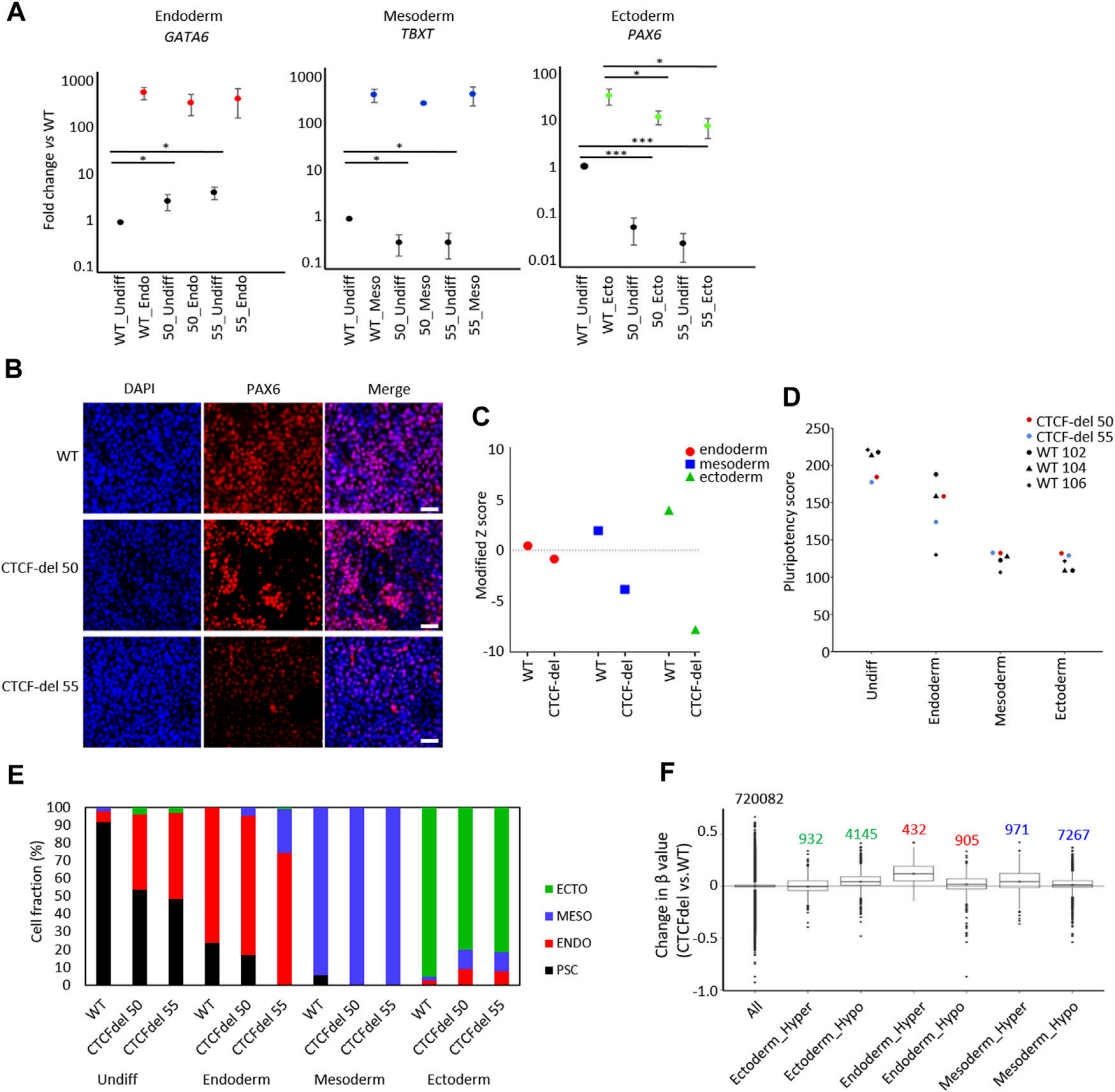
CTCF deletion impairs ectodermal differentiation. **(A)** Expression of marker genes for endodermal (*GATA6*), mesodermal (*TBXT*), and ectodermal differentiation (*PAX6*) were analyzed by qRT-PCR in undifferentiated and differentiated iPSCs (normalized to *GAPDH* and fold-changes *versus* WT; paired t-test ***p-value <0.0005, *: *p*-value <0.05; n = 3). **(B)** Immunofluorescence analysis of PAX6 upon ectodermal differentiation shows marked reduction in CTCF-del cells as compared to WT (nuclei counterstained with DAPI; Scale bar: 50 μm). **(C)** Gene signatures for endoderm, mesoderm, and ectoderm (Schmidt et al., 2023; Zeevaert et al., 2023) were analyzed in undifferentiated WT and CTCF-del iPSCs. Modified Z scores represent normalized expression levels of germ layer specific genes. Ectodermal and mesodermal genes are less expressed in CTCF-del iPSCs. **(D)** Pluripotency score analysis (Schmidt et al., 2023) of WT and CTCF-del iPSC lines indicates slightly lower pluripotent state in CTCF-del cells. **(E)** Deconvolution of undifferentiated and differentiated cells with ‘GermLayerTracker’ (Schmidt et al., 2023). The CTCF-del lines are more primed toward endoderm and their ectodermal differentiation seems to be impaired. **(F)** Box plots indicating the DNA methylation levels in CTCF-del cells compared to WT iPSCs at germ layer specific CpGs (Schmidt et al., 2023). Particularly the CpG-sets hypermethylated in endodermal and mesodermal differentiation revealed higher DNA methylation in CTCF-del iPSCs as well as compared to WT.

To determine the impact of CTCF deletions on directed differentiation of iPSCs, we differentiated WT and CTCF-del lines towards endoderm, mesoderm, and ectodermal lineage. RT-qPCR analysis revealed that already in undifferentiated state, the expression of endodermal gene (*GATA6*) was higher in the CTCF-del lines. However, upon differentiation to the endodermal lineage, the same expression level was reached as in WT lines. In contrast, the ectodermal gene *PAX6* was downregulated in undifferentiated CTCF-del lines, and even after directed differentiation, these clones did not reach the same expression level as WT lines. The mesodermal gene *TBXT* was downregulated in undifferentiated CTCF-del cells but showed similar expression levels as WT upon mesodermal differentiation (Figures 4A,B). To further investigate a bias in differentiation of CTCF-del lines we compared the gene expression signatures for germ layers in the undifferentiated WT *versus* CTCF-del lines. To this end, we used gene sets that were previously shown to be upregulated during lineage-specific directed differentiation (Schmidt et al., 2023; Zeevaert et al., 2023). We observed that genes that are upregulated during ectodermal differentiation were less expressed in the CTCF-del lines before differentiation (Figure 4C). Taken together, these data indicate that in undifferentiated state, as well as during ectodermal differentiation, the CTCF-del cells shift away from the ectodermal lineage, indicating that CTCF plays a role in ectodermal differentiation.

Subsequently, we used established epigenetic biomarkers to estimate pluripotency and differentiation potential of iPSCs based on DNA methylation at specific CpGs. The “Epi-Pluri-Score,” which is used to classify successfully reprogrammed iPSCs from non-pluripotent cells (Lenz et al., 2015), was positive for all WT and CTCF-del iPSC lines (Supplementary Figure S3D). However, when we used the “Pluripotency Score,” which was trained to capture early differentiation events (Schmidt et al., 2023), the results were lower in CTCF-del as compared to WT cells (Figure 4D). Next, we performed targeted DNA methylation analysis at 12 CpGs by pyrosequencing to determine the ‘GermLayerTracker’ signatures (Schmidt et al., 2023). These DNA methylation values can be used for deconvolution to estimate the cell fractions that differentiate toward endoderm, mesoderm, and ectoderm. In the undifferentiated CTCF-del lines, GermLayerTracker predicted an already higher percentage of endodermal cell fraction (42.2% in clone 50% and 48.6% in clone 55). This analysis also indicated that directed differentiation toward mesoderm is enhanced in CTCF-del lines, whereas ectodermal differentiation was reduced, as compared to WT lines (Figure 4E).

To further validate that DNA methylation patterns are indicative of a bias of CTCF-del differentiation potential toward meso-endoderm, we analyzed lineage-specific DNA methylation patterns in the non-differentiated cells. To this end, we used CpGs with significant hyper- and hypomethylation for each germ layer (Schmidt et al., 2023). We analyzed the DNAm levels of 5077 ectoderm-specific CpGs, 8238 mesoderm-specific and 1137 endoderm-specific CpGs. We found CpGs hypermethylated in endoderm and mesoderm were also hypermethylated in CTCF-del cells. This was more prominently seen for endodermal CpGs, validating that CTCF-del iPSCs are pushed towards the endodermal lineage. In contrast, CpGs that are hypomethylated in the ectodermal lineage were hypermethylated in CTCF-del (Figure 4F). Thus, the transcriptional and epigenetic modifications in CTCF-del lines further support the bias toward endo/mesodermal lineages.

## 4 Discussion

Despite being one of the most extensively studied proteins mediating 3D chromatin architecture (Phillips and Corces, 2009), much is unknown about the function of CTCF in development, organogenesis, and differentiation. The embryonic lethality of CTCF null mice and extensive cell cycle defects and apoptosis in tissue-specific CTCF knockouts (Heath et al., 2008; Soshnikova et al., 2010; Moore et al., 2012; Gomez-Velazquez et al., 2017), precludes the investigation of the loss of CTCF in murine development. CTCF depletion in *in-vitro* conditions also exhibit modest transcriptional changes and do not elucidate the role of CTCF in cell fate decisions (Nora et al., 2017; Chen et al., 2019; Kubo et al., 2021). CTCF knockout in Zebrafish embryos also lead to lethality and disorganized chromatin structure as well as dysregulation of developmental genes (Carmona-Aldana et al., 2018; Franke et al., 2021).

To determine the role of CTCF in human iPSC pluripotency and targeted differentiation, we generated CTCF deletion human iPSC lines that show a marked reduction of the CTCF protein but may still express alternative splice variants of CTCF. A recent study (Li et al., 2019) identified a short isoform of CTCF (CTCF-s) that shares 68.8% of the binding sites of the canonical CTCF and can independently bind other target regions. This isoform skips exons 3 and 4 and utilizes a translation start site in exon 5 - thus, our CTCF-del iPSC lines might still express the CTCF-s. The authors also demonstrated that CTCF-s interferes with the normal binding of canonical CTCF and can alter CTCF-mediated chromatin interactions and gene expression (Li et al., 2019). Additionally, CTCF-s was shown to inhibit cell proliferation and promote apoptosis. It may be possible that the binding sites left unoccupied due to the loss of CTCF in our CTCF-del clones were bound by CTCF-s still expressed in these cells, leading to altered gene expression and slower proliferation. CTCF has also been shown to have cell cycle-specific roles in chromatin loop formations (Oomen et al., 2019). Additionally, CTCF is known to regulate cell cycle progression in alpha beta T cells via the regulation of CDK inhibitor transcription (Heath et al., 2008). A similar cell cycle defect could lead to reduced growth in the CTCF-del cells.

Consistent with previous reports on inducible degradation of CTCF (Nora et al., 2017), our CTCF-del lines revealed only moderate differences in gene expression profiles. Furthermore, we did not observe a general trend of global DNA methylation changes, indicating that the effects of CTCF depletion are rather site-specific. CTCF binding sites are considered to be fairly conserved (Kim et al., 2007), and studies have attributed variable binding of CTCF to other factors, including CTCF binding proteins, cell-type specific effects, and DNA methylation. Analysis of certain loci indicates that DNA methylation may hinder CTCF binding at target sequences (Bell and Felsenfeld, 2000; Kanduri et al., 2000; Schoenherr et al., 2003; Wang et al., 2012). CTCF is also known to be associated with methylation-associated silencing of tumor suppressors and oncogenes in immortalized cell lines, where CTCF abrogation is associated with hypermethylated gene promoters (Witcher and Emerson, 2009; Lai et al., 2010; Soto-Reyes and Recillas-Targa, 2010). In contrast, an almost complete loss of DNA methylation still showed retention of 95% CTCF binding in human colorectal carcinoma cell lines (Maurano et al., 2015). Notably, in our CTCF-del lines one of the most extensively studied CTCF targets, *H19*, showed a significant reduction of gene expression accompanied by DNA hypermethylation at 23 CpGs associated with the gene. However overall, the DNA methylation changes in our CTCF-del cells were not generally reflected on transcriptomic level, which is in line with previous reports (Wang et al., 2012). It is conceivable that CTCF has different immediate impacts on transcriptome and methylome at specific sites in the genome. Our analysis also revealed an overlap of 352 differentially methylated CpGs with CTCF peaks. These could potentially be of interest to understand further, the direct correlation between CTCF binding and DNA methylation.

CTCF-del cells showed reduced pluripotency hallmarks and a bias towards the endo-mesodermal lineage with defective ectodermal differentiation. Reports in mESCs indicate that CTCF depletion leads to a downregulation of pluripotency genes and that CTCF is an upstream regulator of OCT4 (Olbrich et al., 2021; Song et al., 2022; Wang et al., 2022). Our data is consistent with these findings albeit not all studied pluripotency genes were downregulated in our clones. This may result from residual CTCF still being expressed in cells or cell-specific differences in CTCF function. A recent study investigated epigenetic and transcriptional changes during the differentiation of human pluripotent stem cells (Madrigal et al., 2023). They observed an enrichment of CTCF binding sites associated with closed chromatin during endodermal differentiation. This may suggest endodermal differentiation is associated with reduced CTCF binding and may explain the endodermal shift we see in CTCF-del cells. CTCF depletion in mESCs resulted in the cells entering a 2C like totipotent state, which is characterized by the upregulation of genes such as *ZSCAN4* and repeats such as MERVL (Olbrich et al., 2021). CTCF-del cells did not show an upregulation of the 2C gene panel that we analyzed. It maybe that the residual CTCF is sufficient to prevent the shift to the 2C state. Notably, ZSCAN4, one of the key upstream genes required for the transition to the 2C state, is already upregulated in CTCF-del cells. Our data therefore suggests that CTCF regulates the human iPSC state by maintaining the pluripotency and differentiation potential on the one hand, and preventing the transition of cells into a totipotent 2C like stage on the other.

It is generally accepted that CTCF regulates chromatin interactions by loop extrusion, which involves formation of chromatin loops between two CTCF bound sites in collaboration with the cohesin complex to permit or insulate long range interactions (Davidson et al., 2023). These interactions play an important role in the progressive establishment of chromatin topology during development and lineage progression and reprogramming (Pekowska et al., 2018; Agrawal and Rao, 2021; Song et al., 2022). Interestingly, the N terminal region of CTCF is shown to constrict the movement of cohesin and hence establish chromatin loops (Pugacheva et al., 2020). Thus, the impaired pluripotency and the altered differentiation potential of the CTCF-del cells maybe a result of aberrant chromatin loop formation resulting from the loss of the N terminal region of CTCF.

Application of human iPSCs in standardized drug-testing regimens as well as regenerative and personalized medicine necessitates validation of pluripotency and fidelity of differentiation (Poetsch et al., 2022). Understanding how differentiation decisions are governed in iPSCs may provide a key to meet these demands. Our results provide additional insights into the role of CTCF in regulating pluripotency and differentiation potential of human iPSCs. The endodermal bias in CTCF-del iPSCs may be utilized to ultimately support directed endodermal differentiation of iPSCs in the future.

## Data Availability

The RNA sequencing, DNA methylation, and chromatin immunoprecipitation sequencing data are 429 available at Genome Omnibus (GEO) under the accession number “GSE247428”.
