# Supplementary material for: CTCF deletion alters the pluripotency and DNA methylation profile of human iPSCs
Source: Front Cell Dev Biol. 2023 Nov 30;11:1302448. doi: 10.3389/fcell.2023.1302448 (PMC10720430; doi:10.3389/fcell.2023.1302448)
Supplement: Supplementary file 1 [file DataSheet1.pdf]

CTCF deletion alters the pluripotency and DNA methylation profile of human iPSCs

Deepika Puri, Catharina Maaßen, Monica Varona Baranda, Kira Zeevaert, Lena Hahnfeld, Annika Hauser, Giulia Fornero, Mohamed H Elsafi Mabrouk, and Wolfgang Wagner

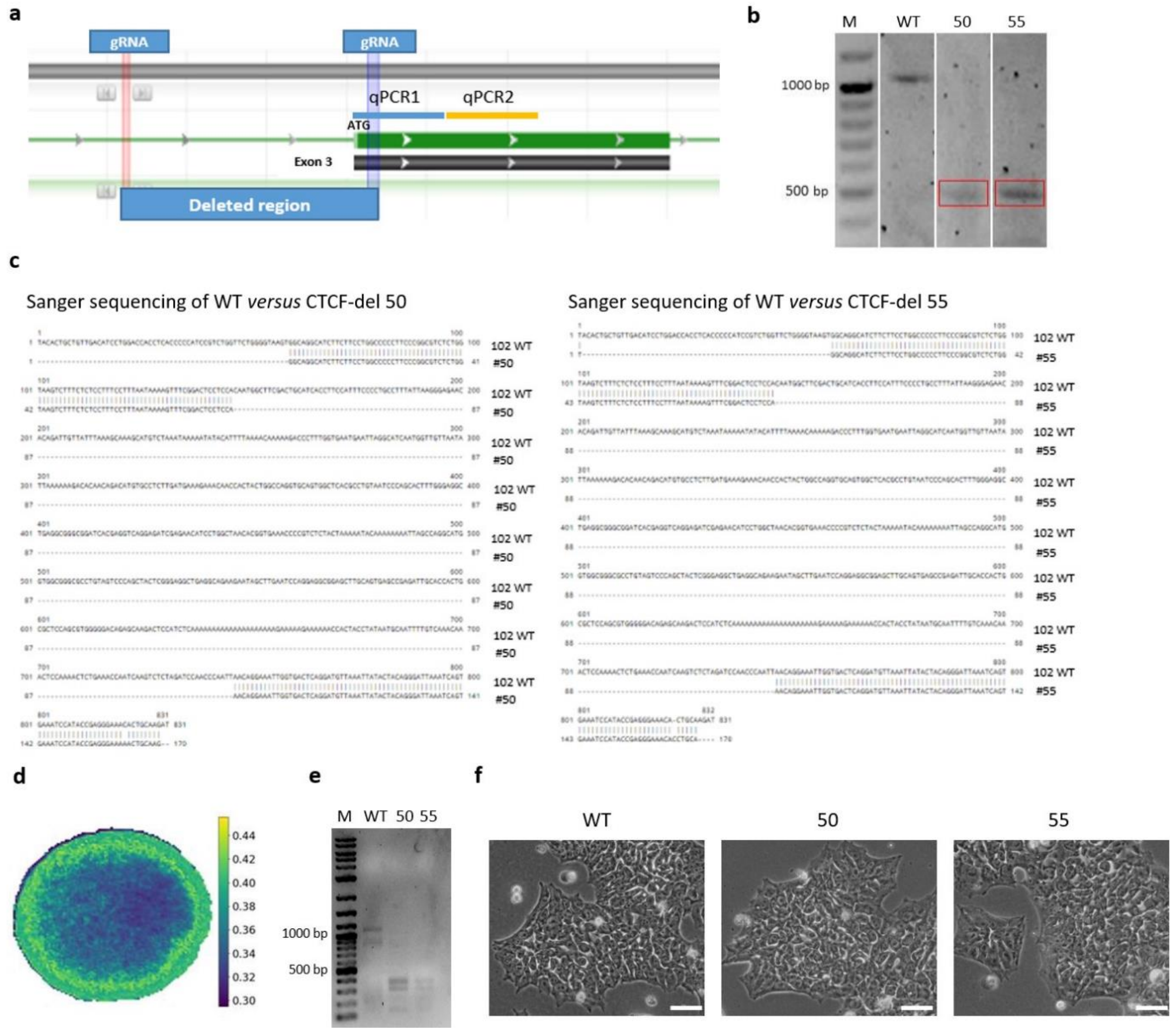

Figure S1: Generation and characterization of CTCF-del iPSCs.

a) CRISPR-Cas9 cloning strategy for CTCF N-terminus deletion. Position of guide RNAs around CTCF exon 3 are indicated in red and blue (AUG = translation start site). The positions of PCR primers for subsequent analysis are also depicted. **b)** Upon gene editing the clones were initially screened with PCR to confirm the loss of a 534bp. This is depicted for the relevant clones (M = marker). **c)** Sanger sequencing analysis of genomic DNA to verify the loss of 534bp including the TSS in CTCF-del clones 50 and 55 compared to WT (syngeneic iPSC line 102). **d)** Spatial reconstruction of normalized gene expression of CTCF based on single-cell RNA seq performed in [1]. **e)** cDNA amplified from WT and CTCF-del cells shows longer transcripts in WT and shorter transcripts in CTCF-del cells. **f)** Brightfield images of WT, CTCF-del 50 and CTCF-del 55 iPSC colonies. No morphological differences were observed in CTCF-del cells (Scale bar: 20µm).

a

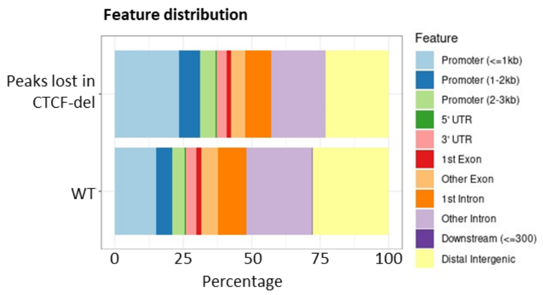

c

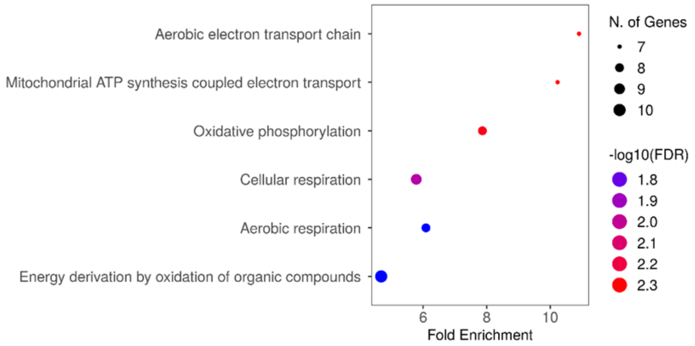

b

Downregulated

gene_name	log2FoldChange	padj
U2AF1	-9.94	9.6E-04
RPL23AP3	-9.63	1.6E-03
HSPA9	-9.50	1.8E-03
SIX3	-9.16	1.8E-05
UVSSA	-8.85	1.3E-03
SNORD3A	-8.73	9.3E-05
PRTG	-8.43	2.1E-05
PPP1R12B	-8.42	3.1E-05
PKN2	-8.41	5.7E-06
CHD9	-8.16	3.2E-05
MATR3	-8.08	4.7E-04
HIST1H1E	-8.06	2.8E-04
ITGB1P1	-7.79	1.4E-02
AC125807.1	-7.78	1.3E-02
SCHIP1	-7.58	4.2E-02
ZNF625	-7.55	7.6E-03
PSMC1P1	-7.54	1.7E-02
IFT81	-7.43	2.5E-03
LINC00884	-7.38	2.2E-04
BCLAF1P2	-7.37	2.2E-02

Upregulated

gene_name	log2FoldChange	padj
TMCC3	8.18	1.38E-03
LINC00689	8.00	2.78E-02
AC005786.1	7.90	3.15E-02
SERTM1	7.79	4.54E-03
AC092807.3	7.51	7.60E-03
NOTUM	6.67	9.98E-03
CALCRL	6.61	5.52E-03
LGALS12	6.60	1.36E-02
AMZ1	6.58	2.68E-02
LCP1	6.54	3.52E-04
IGSF9B	6.41	1.21E-03
LINC00663	6.36	3.81E-02
AC009120.5	6.12	7.25E-03
NDUFV1	6.11	1.16E-03
QPCTL	6.01	2.46E-02
TMEM220	5.84	9.25E-03
PLPPR5	5.61	1.12E-02
NR4A1	5.42	1.42E-02
ARHGAP9	5.31	1.08E-02
SPIB	5.06	4.27E-02

d

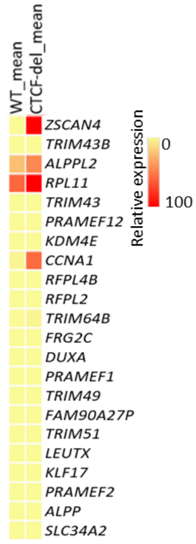

Figure S2: Characterization of upregulated and downregulated genes upon CTCF deletion.
a) Feature analysis of CTCF peaks in WT and peaks lost in CTCF-del cells shows a loss of CTCF peaks in the promoter region **b)** Top 20 downregulated and upregulated genes in CTCF-del cells compared to WT (log2 fold change and adjusted p-values are indicated). **c)** Gene set enrichment analysis of 141 upregulated genes identified by RNA sequencing data of CTCF-del versus WT iPSCs demonstrates significant enrichment in categories for electron transport and aerobic respiration. **d)** Comparison of normalised RNA-seq counts for 2C-specific genes [2] in WT and CTCF-del cells.

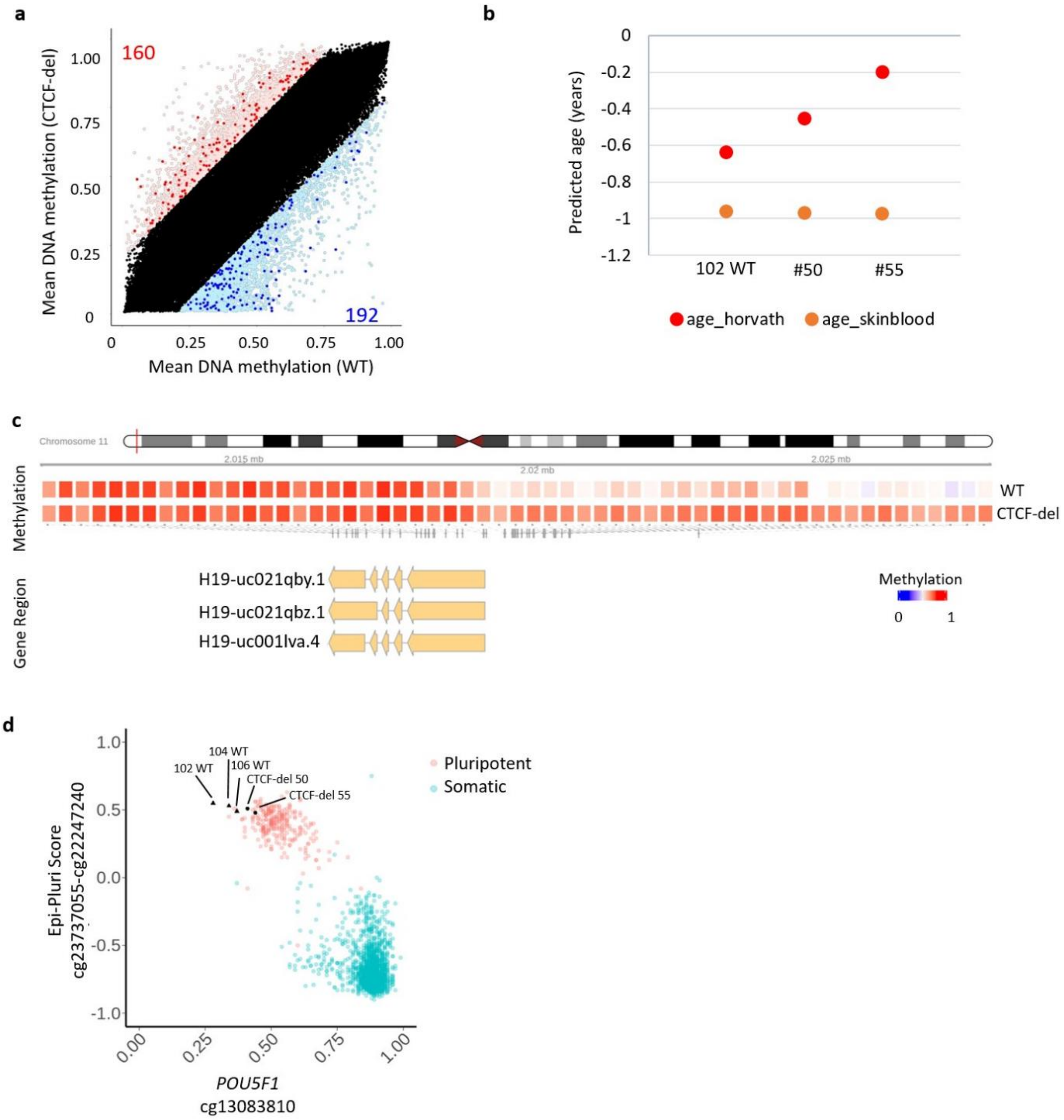

Figure S3: DNA methylation, epigenetic age predictions, and Epi-Pluri-Score analysis of CTCF-del cells.
a) Overlap between hypermethylated (pale red) and hypomethylated (pale blue) CpGs in CTCF-del with CpGs present in CTCF peaks (dark red and blue). **b**) Epigenetic age of WT and CTCF-del iPSCs predicted by the Horvath multi-tissue clock [3] and the Skin-Blood clock [4]. The Y-axis represents predicted age in years. Overall, the predicted ages were always close to zero years, which can be expected for iPSCs. There was no evidence for accelerated epigenetic aging upon CTCF-del. **c**) Methylation analysis of CpGs associated with the *H19* gene shows hypermethylation of CpGs associated with the *H19* promoter region in CTCF-del cells. **b)** Epi-Pluri-Score of WT and CTCF-del cells. This analysis is based on DNAm levels in cg23737055 (*ANKRD46*), cg22247240 (*C14orf115*), and cg13083810 (*POU5F1*) [5]. A positive Epi-Pluri-Score is indicative of pluripotency. The reference clouds refer to DNA methylation profiles of 264 pluripotent (red) and 1,951 non-pluripotent cell preparations (blue) obtained on the Illumina HumanMethylation27 BeadChip platform [5].

Supplemental table 1: Primers used for the study.

**CRISPR-Cas9**		
**Name**	**Target sequence in the genome**	
**crRNA1**	CCTGTTAATTGGGTTGGATCTAG	
**crRNA2**	CCATTGTGGAGGAGTCCGAAACT	
**PCR**		
**Target**	**Forward**	**Reverse**
**CTCF**	ATCTTGCAGTGTTTCCCTCG	TCATGGGTTCACTTTCCGCA
**CTCF cDNA**	TGGACAGATTGCTGACCAGG	CGACGATGCCGAACCAATTC
**RT-qPCR**		
**Target**	**Forward**	**Reverse**
**CTCF1**	CGAAGCCATTGTGGAGGAGT	GTGGCAGGCATCTTCTTCCT
**CTCF2**	CCACTTACCCCAGAACCAGA	GTTTATGGGCTGTTCCTCCA
**TBXT**	CAGTGGCAGTCTCAGGTTAAGAAGGA	CGCTACTGCAGGTGTGAGCAA
**GATA6**	CTCAGTTCCTACGCTTCGCAT	GTCGAGGTCAGTGAACAGCA
**PAX6**	TCGAAGGGCCAAATGGAGAAGAGAAG	GGTGGGTTGTGGAATTGGTTGGTAGA
**POU5F1**	GGGGGTTCTATTTGGGAAGGTA	ACCCACTTCTGCAGCAAGGG
**GAPDH**	GAAGGTGAAGGTCGGAGTC	GAAGATGGTGATGGGATTTC
**Sanger sequencing**		
**Name**	**Sequence**	
**CTCFs**	AGCTGTTCCATCATCACCATCTG	
**Pyrosequencing**		
**Target**	**Forward**	**Reverse**
**SC1**	GGTTGGAGTGTATTGGTGTAA	Biotin-AATCCCAACCTTTATACATATTAAT TCTT
**SC2**	AGGTTGGTTATGAATTTTTGGTTTTAAGTA	Biotin-ATACCCTACCTTCCTTTCATTTATA TTC
**SC3**	GATGTTGAGGGTTAGGGGGTAATT	Biotin-CCTAAAACTCTAAAAATCTTTCTCC CTAAA
**Endo1**	GAATAGTATATGGTTGGTTGGGAAAGT	Biotin-CCAAAAAAAAAAAATACCTTTACT ATCACT
**Endo2**	GGGATGTTGTGGATGGTAAAA	Biotin-ACTCCCACATCTAAACACCTAA
**Endo3**	GGGAGAGGGATTTATTATTAGGT	Biotin-ACCCCCTCCTTCAACTATAAT
**Meso1**	AGGGTAAGGTTGTTTTGTTTAGTTTAT	Biotin-TCATACCTTTAAAACCCACAACTA AAAT
**Meso2**	TGAGTTTGGTTAGTTTAGTTATAGGT	Biotin-CATCCCTAAAACAAACAAAAAAC AATT
**Meso3**	ATGGTTTGGTATAGAAAGTTTATGG	Biotin-ATACTTTCATCTCTTCTAATACCTT TAAC
**Ecto1**	GGGGTTTTGAAAGTAAATGTGT	Biotin-TTCCAACTCACTAAAAAACACTTC
**Ecto2**	AGTGGGAGTAAATGAGTTTAGT	Biotin-CAATTTCAAAATCTCCATCTCAAA ATATCA
**Ecto3**	GGGAGATTTTAGTTTTTTTTGTAGGG	Biotin-CCCAATATTATAATTCTTAACACCT CTCAT

Table continued		
	**Sequencing primers**	
**SC1**	GTTGAGATTATAGGTGTGA	
**SC2**	TTGGGATTATAGGTGTG	
**SC3**	TGAAGGTTTTTTTAGTTTTGA	
**Endo1**	AGGAGTTATTTTATTATATTGGAG	
**Endo2**	AGGGGTGTGGGAAGT	
**Endo3**	GGTTTGAGAAAGAAGTTAG	
**Meso1**	ATTAGGGTTTTGGTTTTATT	
**Meso2**	ATTTGTTGTTGAGGTTTTTAATA	
**Meso3**	GTTTTGTGGGTGGGG	
**Ecto1**	AGTAAATGTGTTGAAAGTT	
**Ecto2**	TTTTAGGGTAAGAAAATATAGATAG	
**Ecto3**	AGTTTTTTTTGTAGGGATTTT	

Supplemental table 2: Antibodies used for the study.

Antibody	Clone	Company	Catalog number	Dilution	Application
**Anti-OCT4**	polyclonal	Abcam	ab19857	1:500	Immunostaining
**Anti-PAX6**	AD2.35	Santa Cruz	sc-53108	1:200	Immunostaining
**Anti-CTCF**	polyclonal	Active Motif	61311	1:2000	Immunostaining
**Anti-CTCF**	polyclonal	Active Motif	61311	1:500	Western blot
**Anti-CTCF**	polyclonal	Active Motif	61311	4µg	ChIP-Seq
**Beta-Actin**	HRP-conjugated Beta Actin Monoclonal antibody	Proteintech	HRP-66009	1:10000	Western blot
**Goat anti-rabbit (Alexa Fluor 594)**		Invitrogen	A-11012	1:200	Immunostaining
**Goat anti-mouse (Alexa Fluor 594)**		Invitrogen	A-11032	1:200	Immunostaining

Supplemental References

1. Mabrouk, M.H.E., et al., *The spatial self-organization within pluripotent stem cell colonies is continued in detaching aggregates.* Biomaterials, 2022. **282**: p. 121389.
2. Hendrickson, P.G., et al., *Conserved roles of mouse DUX and human DUX4 in activating cleavage-stage genes and MERVL/HERVL retrotransposons.* Nature Genetics, 2017. **49**(6): p. 925-+.
3. Horvath, S., *DNA methylation age of human tissues and cell types.* Genome Biol, 2013. **14**(10): p. R115.
4. Horvath, S., et al., *Epigenetic clock for skin and blood cells applied to Hutchinson Gilford Progeria Syndrome and ex vivo studies.* Aging (Albany NY), 2018. **10**(7): p. 1758-1775.
5. Lenz, M., et al., *Epigenetic Biomarker to Support Classification into Pluripotent and Non-Pluripotent Cells.* Scientific Reports, 2015. **5**: p. 8973.
